# Overweight and aging increase the risk of atrial fibrillation after cardiac surgery independently of left atrial size and left ventricular ejection fraction

**DOI:** 10.1186/s13019-020-01366-x

**Published:** 2020-10-15

**Authors:** Pier Luigi Stefàno, Marco Bugetti, Guido Del Monaco, Gloria Popescu, Paolo Pieragnoli, Giuseppe Ricciardi, Laura Perrotta, Luca Checchi, Roberto Rondine, Sergio Bevilacqua, Carlo Fumagalli, Niccolò Marchionni, Antonio Michelucci

**Affiliations:** 1 Department of Cardiothoracovascular Medicine, Careggi University Hospital (AOUC), Largo Brambilla, 3, 50134 Florence, Italy; 2 Department of Experimental and Clinical Medicine, University of Florence, Largo Brambilla, 3, Florence, 50134 Italy

**Keywords:** Postoperative atrial fibrillation, Cardiac surgery, Risk factors

## Abstract

**Background:**

Body mass index (BMI), age, left atrium (LA) dimension and left ventricular ejection fraction (LVEF) have been linked to post-operative atrial fibrillation (POAF) after cardiac surgery. The aim of this study was to better define the role of these risk factors.

**Methods:**

This retrospective cohort study evaluated 249 patients (without prior atrial dysrhythmia) undergoing cardiac or aortic surgery. Prior to surgery, the following data were collected: age, BMI, LA diameter, LA area, LVEF, thyroid stimulating hormone (TSH), creatinine and the presence of arterial hypertension (AH) and diabetes. Intraoperative data such as operation time, total clamp time, cardiopulmonary bypass time, and presence of pericardial/pleural effusion were also collected. Only patients without pre- and post-surgery prophylactic anti-arrhythmic therapy were included.

**Results:**

Patients with (*N* = 127, 51%) and without POAF (*N* = 122, 49%) were compared. No difference was observed for sex, LA diameter, LA area, LVEF, TSH, diabetes and use of ACE inhibitors or statins prior to intervention. Moreover, no difference was observed in terms of operation time, total clamp time, cardiopulmonary bypass time, and presence of pericardial/pleural effusion. However, patients with POAF were older (70.6 ± 10.7 vs. 60.4 ± 16.4 years, *p* = 0.001), had higher BMI (26.8 ± 4.5 vs. 24.9 ± 3.6 kg/m^2^, *p* = 0.001), higher baseline creatinine (1.06 ± 0.91 vs. 0.88 ± 0.32 mg/dL, *p* = 0.038) and a higher frequency of arterial hypertension (73.2% vs. 50%, *p* = 0.001) and Bentall procedure (24.4% vs. 9.8%, *p* = 0.023). Multivariate analysis showed that the only independent predictors of POAF were age (OR = 1.05, 95%CI 1.02–1.07, *p* = 0.001) and BMI (OR = 1.11 95%CI 1.03–1.2,*p* = 0.006).

**Conclusions:**

These findings suggest that advanced age and a higher BMI are strong risk factors for POAF in patients without previous AF even in the presence of comparable LA dimensions and LVEF.

## Introduction

Because of persistent high incidence, increased complications, length of hospitalization and costs, post-operative atrial fibrillation (POAF) is still considered a major problem in cardiac surgery [1–4]. It is therefore useful to determine which clinical and instrumental factors may identify patients at increased risk of developing POAF.

A close association has been reported between obesity and atrial fibrillation [5]. However, almost all studies reporting this association have used the body mass index (BMI) to evaluate weight excess [6]. Indeed, BMI is still considered the most practical parameter taken to represent the role played by overweight [7], while other recognized risk factors for POAF include advanced age [8, 9], left atrial (LA) dimension [10, 11] and left ventricular systolic dysfunction [12, 13].

The aim of the present study was to better define the relative role played by these risk factors comparing patients with and without POAF. To eliminate the role of arrhythmia itself as a confounder, only patients without a previous history of AF were included in the present study.

## Methods

### Study population and data collection

This retrospective cohort study undertaken in 2019 included 249 consecutive patients (mean age 65.6 ± 14.7, range 19–90 years, and 62.7% were male) who underwent cardiac or aortic surgery in a tertiary hospital in Florence, Italy. Patients undergoing emergency surgery, and those with prior atrial dysrhythmia (based on clinical history and review of medical records), were excluded. All surgical procedures were performed in extracorporeal circulation and in cardioplegic arrest. In no case was the left auricle closed or removed or surgical ablation of atrial fibrillation performed. The following data were systematically collected 24 h before surgery: age, BMI, left atrium (LA) diameter, LA area, left ventricular ejection fraction (LVEF), thyroid stimulating hormone (TSH), creatinine and the presence/absence of arterial hypertension (AH) and diabetes. To detect the occurrence of incident POAF, cardiac rhythm was continuously recorded over the first seven days postoperatively. The end-point of the study was the occurrence of POAF during the early postoperative period (i.e. the time between surgery and discharge, defined as any sustained; i.e. > 10 min, recorded episodes [6, 8]). The presence of pleural and pericardial effusion were evaluated postoperatively. Moreover, operation times, clamp times, and cardiopulmonary bypass times were also measured. No pre- and post-surgery therapies were used to prevent POAF. Amiodarone and/or electrical cardioversion were used to stop POAF.

This study received approval from the institutional review board of the hospital for accessing the electronic medical records of study subjects, and for collecting and processing relevant data from these records. The research plan was submitted to the department of cardiovascular surgery and nursing departments to report the purpose and method of the study and to obtain permission for data collection. This investigation conformed to the principles outlined in the Declaration of Helsinki.

### Statistical analysis

Continuous variables were reported as mean ± standard deviation (SD) whereas categorical variables were presented as number and percentages. All variables were compared for the presence of POAF (‘no’ vs. ‘yes’). For continuous variables, comparisons were performed using t-test, analysis of variance or nonparametric tests, as appropriate. Categorical variables were compared using the Chi-squared test, or Fisher’s exact test when any expected cell count was less than five. Multivariate logistic regression analysis (method selection: backward deletion) was used to identify independent predictors of incident POAF. In this regard, all variables with p < 0.10 following univariate analysis were included in the model. In addition, potential candidate variables with *p* > 0.010 but with potential impact on the pathophysiology of POAF were also entered in the model. All statistical analyses were performed with SPSS V23 package (SPSS Inc., Chicago, IL, USA).

## Results

The main demographic, clinical, laboratory, echocardiographic characteristics, and the surgical procedures of patients without and with incident POAF are presented in Table 1.

**Table 1:** Demographic and clinical characteristic in patients without and with incident POAF

	**Incident POAF**		
**Variable**	**No** **(***N*** = 122)**	**Yes** **(*N* = 127)**	***P*-value**
Age, (mean ± standard deviation) years	60.4 ± 16.4	70.6 ± 10.7	0.001
Male, n (%)	79 (64.8%)	77 (60.6%)	0.59
BMI, (mean ± standard deviation) kg/m^2^	24.9 ± 3.6	26.8 ± 4.5	0.001
Arterial hypertension, n (%)	61 (50%)	93 (73.2%)	0.001
Diabetes, n (%)	17 (13.9%)	23 (18.1%)	0.37
Creatinine, (mean ± standard deviation) mg/dL	0.88 ± 0.32	1.06 ± 0.91	0.038
TSH, (mean ± standard deviation) mU/L	2.69 ± 4.33	1.96 ± 1.69	0.13
ACE-I, n (%)	43 (35.2%)	47 (33.8%)	0.85
Statins, n (%)	27 (22.2%)	39 (30.7%)	0.15
LA diameter, (mean ± standard deviation) mm	39.2 ± 8.1	40.1 ± 10.1	0.70
LA area, (mean ± standard deviation) cm^2^	23.9 ± 5.9	23.6 ± 5.9	0.69
LVEF, (mean ± standard deviation) %	58.6 ± 9.8	57.6 ± 7.9	0.41
CABG, n (%)	20 (16.4)	31 (24.4)	0.12
Operation time, (mean ± standard deviation) minutes	212 ± 74	227 ± 100	0.16
Clamp time, (mean ± standard deviation) minutes	70 ± 34	76 ± 32	0.13
Cardiopulmonary bypass time, (mean ± standard deviation), minutes	97 ± 51	101 ± 43	0.56
Mitral valve repair, n (%)	31 (25.4)	28 (22.0)	0.53
Tricuspid valve repair, n (%)	10 (8.2)	8 (6.3)	0.56
Aortic valve replacement, n (%)	49 (40.2)	50 (39.4)	0.90
Mitral valve replacement, n (%)	12 (9.8)	9 (7.1)	0.44
Tricuspid valve replacement, n (%)	1 (0.8)	0	0.49
Morrow septal myectomy, n (%)	3 (2.5)	5 (3.9)	0.72
Atrial septal defect closure, n (%)	4 (3.3)	3 (2.4)	0.72
Ascendant aorta replacement, n (%)	10 (8.2)	9 (7.1)	0.74
Bentall procedure, n (%)	12 (9.8)	31 (24.4)	0.023
Ventricular septal defect closure, n (%)	2 (1.6)	0	0.24
Pericardial effusion, n (%)	36 (29.8%)	35 (27.3)	0.67
Pleural effusion, n (%)	34 (28.1%)	33 (26.0%)	0.68

*POAF* Post-operative atrial fibrillation, *BMI* Body mass index, *TSH* Thyroid stimulating hormone, *ACE-I* Angiotensin Converting Enzyme inhibitors, *LA* Left atrium, *LVEF* Left ventricular ejection fraction, *CABG* Coronary artery bypass graft

**Fig. 1:**
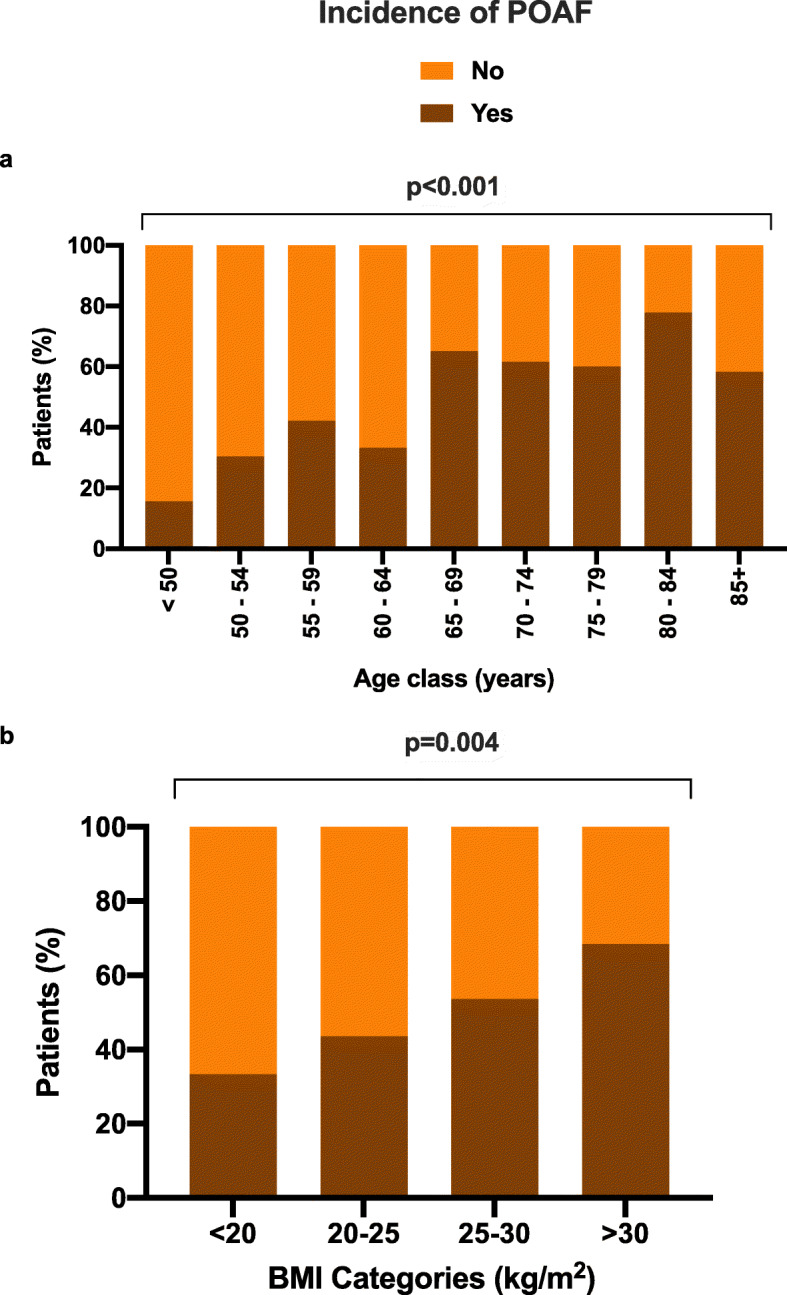
Increase in incidence of post-operative atrial fibrillation (POAF) by age-class (**a**) and by body-mass index (BMI) category (**b**). *P*-values denote statistically significance differences across age and BMI categories respectively

The incidence of POAF was observed to increase with advancing age and increasing BMI (Fig. 1). Overall, patients with incident POAF were significantly older (70.6 ± 10.7 vs. 60.4 ± 16.4 years, *p* = 0.001) and had significantly higher BMI (26.8 ± 4.5 vs. 24.9 ± 3.6 kg/M^2^, *p* = 0.001) and baseline creatinine (1.06 ± 0.91 vs. 0.88 ± 0.32 mg/dL, *p* = 0.038) as well as a greater prevalence of history of arterial hypertension (73.2% vs. 50%, *p* = 0.001) (Table 1). No other laboratory data or echocardiographic parameter, including left atrial dimensions or LVEF emerged as being significantly different between the two groups. Among surgical procedures, only the Bentall operation was performed much more frequently in patients with POAF compared to those without incident POAF (24.4% vs. 9.8%, *p* = 0.023) (Table 1).

Multivariate analysis with a binary logistic model included the following covariates as variables associated with POAF that remained significant after univariate analysis: BMI, arterial hypertension, plasmatic creatinine and Bentall procedure in addition to the following variables: use of ACE-I, statins, operation time, total clamp time, cardiopulmonary bypass time, and presence of pericardial/pleural effusion. Considering all these variables, only age (OR = 1.05, 95% CI 1.02–1.07, *p* = 0.001) and BMI (OR = 1.11, 95%CI 1.03–1.2, *p* = 0.006) were emerged as independent predictors of incident POAF.

## Discussion

Using a real-world clinical experience with careful exclusion of patients with a history of atrial tachyarrhythmia, the results of this study provide solid evidence that advanced age and elevated BMI can be considered important POAF risk factors in patients who never had experienced AF prior to surgery. In fact, the association of these two parameters with the probability of incident POAF proved to be independent of left atrial size and ventricular systolic function, as well as the type of surgery performed.

Some previous studies have attempted to analyze the behaviour of POAF risk factors in patients without presurgical AF, as in the present study. However, they showed some differences. Sun et al. [14] analyzed only CABG patients and did not evaluate the predictive value of atrial dimensions. Serban et al. [15], defined differently POAF and did not find a relationship between POAF and age. Moreover, and in contrast to the present study, all patients received an oral beta blocker during the postoperative period, starting with a dose of 25 mg twice daily. Bramer et al. [16] identified BMI and age as POAF risk factors without analyzing atrial dimensions and even in this case prescribing postoperatively a betablocker (metoprolol) as POAF prophylaxis. The present study analyzed multiple risk factors without the use of prophylactic therapy and we have shown that BMI and age can predict POAF independently of ejection fraction and atrial dimension.

Advanced age has been defined as one of the most powerful risk factors for incident AF following open heart surgery [8, 9]. This may be linked the presence of pre-existing structural changes of the atria related to ageing and to arterial hypertension.

A BMI beyond the diagnostic threshold of obesity suggests that a low-grade inflammation might be present in patients with POAF [17], and excess fat, particularly epicardial fat, is known to contribute to structural alterations of the atrial tissue [18]. As in previous studies [6, 8], we defined POAF as episodes of AF lasting longer than 10 min and/or entailing the use of antiarrhythmic therapy or electrical cardioversion. This definition allows the possibility to identify POAF even when continuous monitoring is interrupted. Current evidence suggests that the vast majority of episodes still occur in the first 3–4 days and are generally sustained for more than an hour. Consequently, the risk of unrecognized episodes would be particularly low.

## Conclusions

Age and body mass index represent important risk factors for POAF independent of atrial size and ventricular systolic function.

## Data Availability

Not applicable.

## Abbreviations

AH: Arterial hypertension
BMI: Body mass index
LA: Left atrium
LVEF: Left ventricular ejection fraction
POAF: Postoperative atrial fibrillation
TSH: Thyroid stimulating hormone
